# Differences between adolescents exhibiting moderate binging and non-binging eating behaviors

**DOI:** 10.1186/s40064-015-1372-1

**Published:** 2015-10-13

**Authors:** Francesca Cuzzocrea, Sebastiano Costa, Rosalba Larcan, Mary Ellen Toffle

**Affiliations:** Department of Human and Social Sciences, University of Messina, Via Tommaso Cannizzaro, 278, Messina, Italy; Department of Law and History of Institutions, University of Messina, Piazza XX Settembre, 4, Messina, Italy

**Keywords:** Adolescence, Personality, Eating behavior, Binge eating

## Abstract

Much research has been conducted to study the association between personality and eating disorders using clinical samples. However, less research has been done on personality variables in non-clinical cases of adolescents prone to binge eating. The purpose of this study is to compare a group of 53 adolescents without binge eating with a group of 28 adolescents with moderate binging behaviors and to investigate the relationship between personality traits and eating behaviors. All participants completed BES, STAY, EPQ-R, IVE and EDI-2. The results demonstrated that the group with moderate binging presented higher scores in state and trait anxiety, psychoticism, neuroticism, and impulsivity than the adolescents without binge eating. The second hypothesis of this research was to analyze the relationship between personality characteristics and eating behaviors. In the group of adolescents without binge eating both neuroticism and psychoticism correlated with ED symptomatology. Similarly extraversion, impulsivity and venturesomeness correlated with ED symptomatology. In the group of adolescents with moderate binge eating, there was an association of trait anxiety, extraversion, venturesomeness and empathy with ED symptomatology in university samples. The results of this study represent a new stimulus to thoroughly investigate those aspects of personality that may be predictive of ED symptomatology and to develop preventative strategies. It is our opinion that it is necessary to focus attention not only on clinical or non-clinical samples, but also on adolescents who could be considered at risk.

## Background

Binge-eating disorder (BED) is characterized by recurrent episodes of excessive food consumption with experienced loss of control and marked distress but without the inappropriate compensatory behaviors of bulimia nervosa (BN) (Marcus and Wildes 2013). The literature demonstrates that binge eating is associated with several negative health outcomes including obesity, mental disorders, suicide attempts, and impairment in professional, personal, and social domains (Hudson et al. 2007; Wonderlich et al. 2009). Marcus and Wildes (2013) stated that the exact causes of binge eating disorder (BED) are unknown, even though research has identified several potential biological and environmental risk factors. The literature demonstrates the relevance of multiple factors: biological, psychological and social factors have been implicated in the pathogenesis of BED (Lanzarone et al. 2014). Previous cross-sectional studies (Gordon et al. 2012; Kleifield et al. 1994; Bulik et al. 1995) suggest that for identifying at-risk populations, it is important to identify the personality traits that individuals with disordered eating demonstrate. However, to better understand the protective factor for BED it could be relevant to explore also the characteristics of the subjects that do not demonstrate any symptoms of BED. For this reason the aim of this explorative study is to explore the characteristics that distinguish individuals with a risk of developing BED from those who do not.

There is a wide range of scientific literature that proposes an explanation of the relationship between eating disorders and personality characteristics. However, major studies have looked primarily at clinical sampling and in particular have studied adolescents diagnosed with anorexia nervosa and bulimia nervosa. For this reason, it is difficult to summarize the characteristics of risk and protective factor for BED. The review proposed by Pearlstein (2002) demonstrated how the characteristics of clinical patients were heterogeneous although some overlapping exists in that risk factors cannot be transmitted across subtypes of eating pathologies.

This paper analyzes the relationship between personality traits and eating behaviors with selected non-clinical samples. Subjects who did not present all criteria for a diagnosis of BED, but demonstrated some potentially risky eating behaviors were examined. In order to define which personality traits enter into the analysis of eating behaviors, many researchers have referred to the Eysenck and Eysenck theory model (Eysenck et al. 1985; Eysenck and Eysenck 1993). Eysenck and Eysenck (1985, 1993) define personality as the interaction between three central traits: extraversion, neuroticism, and psychoticism. Extraversion is linked to a low inner arousal level. People high in neuroticism are seen as emotionally unstable whereas people low in neuroticism are seen as being emotionally stable. Psychotic people tend to be aggressive, cold, egocentric, and impulsive. As summarized by Cassin and von Ranson (2005), many studies have shown that the association between psychoticism and ED symptomatology is not clear (Finlayson et al. 2002; Geissler and Kelly 1994; Janzen et al. 1993; Diaz-Marsa et al. 2000). Many studies, instead, consistently associate neuroticism with eating disorders: individuals with eating disorders score higher on neuroticism than controls (Podar et al. 1999; Brookings and Wilson 1994; Claridge and Davis 2001). Furthermore, Cassin and von Ranson (2005) reported that the studies conducted before 2005 did not confirm the association between extraversion and eating behaviors in both clinical (anorexia and bulimia) (Podar et al. 1999; Brookings and Wilson 1994) and non-clinical samples (Claridge and Davis 2001; Gual et al. 2002). However, other studies of clinical adolescents found low levels of extraversion to be related to disordered eating (anorexia and bulimia) (De Silva and Eysenck 1987; Feldman and Eysenck 1986). Similarly, Bruce et al. (2004) linked bulimia nervosa to social avoidance in a clinical sample of 59 women aged 18–45. Exceptions to these studies are findings by Wade et al. (1995) who reported no relation between extraversion and bulimia nervosa in clinical samples.

Eysenck and Eysenck (1985, 1993) define some second order traits that constitute the three above-mentioned principal traits. However the literature has focused less on the role of these components as impulsivity (Wonderlich et al. 2004; Stojek et al. 2014) and anxiety (Goldfield and Legg 2006; Bardone-Cone et al. 2013) in eating behaviors. For instance, the relation between impulsivity and eating behavior in a clinical sample (Claes et al. 2005) and in a non-clinical sample (Lyke and Spinella 2004) was examined, in cross-sectional studies, showing positive relationships. According to Cassin and von Ranson (2005) impulsivity may be attributed to the erratic dietary patterns and emotional instability associated with bulimia. The authors concluded that a better understanding of the personality type that experiences various types of disordered eating in association with anxiety is necessary in order to propose possible focused intervention.

As summarized by Cassin and von Ranson (2005), most research has studied the association between personality and eating disorders focusing on anorexia nervosa and bulimia nervosa, while less research has been done on personality variables in patients who are prone to BED. Dahla et al. (2012) indicated that obese patients with relevant eating disorders have more anxiety, depression, and neuroticism than obese patients without eating disorders. The findings of a previous study (Bulik et al. 2002) suggest that more neurotic personalities are found among obese patients with eating disorders.

Few studies have focused on samples of non-clinical adolescents. Miller et al. (2006) reported the interaction of neuroticism and extraversion in relation to symptoms of eating disorders in a non-clinical sample of university students. Keel et al. (1998) indicated that adolescent males who reported disordered eating (anorexia and bulimia) expressed greater body dissatisfaction, depression, restraint, and poorer interoceptive awareness as compared to matched and randomly selected controls. More recently, Fitzsimmons-Craft et al. (2012) examined, the relation between reports of anxiety, dimensions of trait perfectionism and BED in non-clinical university students in a longitudinal study. They support the notion that BED is generally affected by both trait- and state-like characteristics. Similar results were found in a cross-sectional study by Cuzzocrea et al. (2012) who reported that these aspects seem to play a role in eating behaviors in non-clinical adolescents: neuroticism seems to be the personality trait most related both in males and females, while psychoticism seems to better differentiate eating behaviors between genders.

While preliminary research supports the role of personality traits in relation to disordered eating in non-clinical and clinical populations, it remains unclear how this aspect is related to the risk of developing a problem eating condition. In our opinion, in order to improve our understanding of how multiple factors interact to determine the onset and maintenance of BED, it could be useful to compare “at risk” individuals with individuals without BED. In fact, few studies have focused on samples of adolescents “at risk”, even if the literature has confirmed an increased risk of adolescents with eating disorders (Lal and Abraham 2011). Furthermore a relevant aspect that is underestimated in previous research is the study of subjects that do not demonstrate symptoms of BED. Understanding the factors that could characterize subjects without problems of BED and subjects that are at risk, could have a relevant role in developing preventive and intervention programs.

This research analyses the relationship between personality traits and eating behaviors in adolescents with moderate binging or no binging. The first aim was to verify whether there are differences between adolescents (moderate binging vs. non-binging) in regards to eating behaviors, personality traits, and state and trait anxiety.

The second aim was to analyze the relationship between eating behaviors and personality traits in two groups of adolescents, in order to identify the correlation between variables: one with moderate binging and one without binging. In particular, we studied whether personality traits and state and trait anxiety can be related to eating behaviors as differentiated in these groups.

### Hypotheses

1. Moderate binging adolescents will show a higher level of eating disorder symptoms, psychoticism, neuroticism, impulsivity, venturesomeness, state anxiety and trait anxiety, than non- binging adolescents.
2. A different pattern of correlation will be shown in the two groups (moderate binging vs. non-binging) of adolescents between personal characteristics (personality traits, and state and trait anxiety) and eating disorder symptoms.

## Methodology

### Participants

A group of eighty-one (81) adolescents was selected to satisfy the inclusion criteria and was categorized in two groups. Fifty-three (53) adolescents (32 males and 21 females; age: M = 16.98, SD = .89; BMI: M = 18.90, SD = .78) were placed in the group of adolescents without binge eating, and 28 individuals (15 males and 13 females; age M = 17.36, SD = .99; BMI: M = 19.10, SD = .82) were included in the moderate binging group. Preliminary analyses showed no difference between groups for age [*t*(79) = 1.74; p = .19], and gender (χ^2^ = .35, p > .05) and BMI [*t*(79) = 1.08; p = .28]. All subjects belonged to a middle socio-economic level. All participants provided self reports of current weight (kg) and height (m), and body mass index was calculated. In the current sample, all adolescents had a normal weight and no one exceeded the EPQ lie scale cutoff score.

### Procedure

Individuals were recruited for voluntary participation from three different high schools in Messina (Italy) that were particularly focused on the prevention of eating disorders. This initial screening was later used to organize a focus group on these topics and those who participated in this first phase had the opportunity to follow specific courses of psycho-education on eating disorders. All participants provided informed consent before participation. If the participants were less than 18 years of age, their parents signed an informed consent. Participants were asked to fill in the Italian version of three questionnaires, individually presented in the classroom context. The order was balanced within groups.

The Binge Eating Scale (Gormally et al. 1982) was completed by 631 individuals (ages: 15–22; 17.5 ± 1.42) to categorize participants into binge eating and non-binge eating groups. A score of 0–17 was considered “absent”; 18–26 “moderate”; and ≥27 “severe” (Dalle Grave et al. 2010). In accordance with the standard cut-off point (Dalle Grave et al. 2010), adolescents who had obtained a score of 0 on the BES were included in the group of adolescents without binge eating, while adolescents who obtained a score from 18 to 26 on the BES were included in the group of moderate binging. A group of adolescents without binge eating was formed by selecting only those who had obtained a score equal to zero, while the group of moderate binging was formed by selecting only those subjects who obtained a score from 18 to 26. Most of the participants had scored on the BES between 0 and 17. However for research purpose, only subjects who had a score of 0 (zero) were selected. The inclusion criteria for the group without binge eating group was very strict (selecting only those subjects who obtained a score equal to 0) in order to better understand the psychological characteristics of individuals with absolutely no risk of developing BED.

Group meetings with a psychologist were immediately proposed for those participants (15 males and 13 females) who obtained scores from 18 to 26, while individual meetings were organized for those (4 females and 3 males) who obtained a score higher than 27. All subjects with severe binging were excluded from this research. However, these subjects were offered psychological support.

### Instruments

1. The *State*-*Trait Anxiety Inventory* (*STAI*-*Y*) [Italian version of Spielberger (1989)] is a 40-item instrument for measuring anxiety in adults on a frequency Likert Scale ranging from 1 (not at all) to 4 (almost always). It clearly differentiates between the temporary condition of “state-anxiety” (20 items) and the more general and long-standing quality of “trait-anxiety” (20 items). Accordingly, sub-scale scores ranged from 20 to 80. Previous studies have shown good levels of reliability and validity for this measure (Spielberger 1989).
2. *Eysenck Personality Questionnaire*-*Revised* (*EPQ*-*R*) Italian version (Eysenck and Eysenck 2004). Participants were asked to complete the 48-item short-scale version of the EPQ-R, which measures four major personality dimensions: *Psychoticism*, *Neuroticism*, *Extraversion* and *Social Desirability*/*Lie Scale*. Participants were asked to rate each item with “yes” or “no” (coded as 1 and 0) depending on how applicable the statement was to them. Accordingly, sub-scale scores ranged from 0 to 12. The psychometric characteristics of this measure have been widely verified in previous studies (Eysenck and Eysenck 2004).
3. The Italian version of the *Impulsivity questionnaire*-*IVE* (Eysenck and Eysenck 2004) was administered in order to assess impulsivity (19 items), venturesomeness (16 items), and empathy (19 items). It is a self-reporting questionnaire composed of 54 items in a yes/no format (coded as 1 and 0) that yields three independent scores which range from 0 to 19 for impulsiveness and empathy, and from 0 to 16 for venturesomeness. High scores indicated high levels of these personality characteristics. Previous studies verified reliability and validity for this measure (Eysenck and Eysenck 2004).
4. *Eating Disorder Inventory*-*2* (EDI-2) (Garner 1995) was used for self-assessment of eating disorder symptoms. It is made up of 64 questions grouped into 11 scales: a self-report measure consisting of 11 subscales that assess specific cognitive and behavioral dimensions of eating disorders: drive for thinness, bulimia, body dissatisfaction, ineffectiveness, perfectionism, interpersonal distrust, interoceptive awareness, maturity fears, asceticism, impulse regulation, and social insecurity. For each item, the participants had to answer “always”, “usually,” “often,” “sometimes,” “rarely”, or “never”. The rating is measured in a range between 0 and 3. The sub-scale scores are calculated by simply adding all the scores of items of each specific sub-scale. This questionnaire has been shown to have good internal consistency, test–retest reliability, 36 as well as validity (Garner 1995).
5. *Binge Eating Scale* (Gormally et al. 1982) is a sixteen-item questionnaire used to assess the presence of binge eating behavior indicative of an eating disorder. Each item has three or four weighted statements, and the respondent is directed to choose one. Respondents indicate which statement is most similar to their eating, and the measure is scored by summing the weighted scores for each item with the possible range of scores from 0 to 46. The Binge Eating Scale has also been used in normal-weight samples (Dalle Grave et al. 2010) to categorize participants into binge eating and non-binge eating groups. In this research the total score was used to yield a continuous measure of binge eating tendencies. The scale has been validated with treatment-seeking obese individuals and has demonstrated high internal consistency (Gormally et al. 1982).

### Data analysis

Four Profile Analysis MANOVA were performed on subjects with the last factor examining whether moderate binging and no-binging adolescents differ on: state and trait anxiety (STAY), major personality trait (EPQ), second order personality trait (IVE), and eating behaviors (EDI-2). Specifically four Profile Analysis were conducted to compare two groups measured on several different scales, all at one time (Tabachnick and Fidell 2013). This is a multivariate equivalent of repeated measures and is a special application of multivariate analysis of variance (MANOVA) to a situation where there are several DVs, all measured on the same scale (or scales with the same psychometric properties) (Tabachnick and Fidell 2013). All the score were converted in T scores, using the norms in the manual of the instruments, to allow comparison between different scales. However, before running the four MANOVA, parametric assumptions on data (linearity, collinearity, and multivariate normality) were assessed by inspecting diagnostic plots and performing ad hoc statistical tests such as the Shapiro–Wilk test for multivariate normality. Although several assumptions were more or less violated, MANOVA is robust enough to deal with such violations (Tabachnick and Fidell 2013). Box’s M was used to assess the homogeneity of variance, suggesting the homogeneity of the covariance matrices regarding the two groups considered in the study for the anxiety scales, first order personality traits, and second order personality traits. A significant value at Box’s M test was found for the eating behaviors. However the eating behaviors comprised many subscales and according to Tabachnick and Fidell (2013), when there are many DVs and a great discrepancy between cell sample sizes, then there is more potential for distortion of the alpha levels. A post hoc test performed with Bonferroni adjustments was used for simple comparisons. Pearson correlations were used to verify the interrelations between study variables. Finally, in order to examine whether personality traits are related to eating behavior, separate hierarchical multiple regression analyses were carried out.

## Results

### Differences between and within groups

Table 1 synthesizes the means, standard deviations and internal consistency (Cronbach α) of scores obtained by adolescents with non- binging and moderate binging.

**Table 1:** Descriptive analyses

	Moderate binging		Without binging	
	M (SD)	α	M (SD)	α
Anxiety (STAY)				
State-Anxiety	57.62 (12.04)*	.89	46.06 (8.41)	.90
Trait-Anxiety	57.44 (11.82)*	.92	44.35 (9.76)	.91
Personality (EPQ and IVE)				
Psychoticism	54.48 (14.29)*	.51	49.10 (9.23)	.50
Extraversion	53.55 (8.41)	.72	53.02 (9.08)	.70
Neuroticism	58.89 (9.53)*	.64	49.04 (9.74)	.61
Lie	44.05 (8.10)	.55	53.72 (8.68)*	.62
Impulsivity	65.33 (10.33)*	.59	52.43 (10.69)	.63
Venturesomeness	55.35 (7.58)	.71	51.69 (9.23)	.73
Empathy	54.46 (10.40)	.76	53.37 (10.10)	.78
Eating disorders (EDI-2)				
Drive for thinness	59.13 (13.41)*	.89	43.58 (3.10)	.85
Bulimia	63.92 (14.97)*	.63	46.96 (9.03)	.52
Body dissatisfaction	54.85 (11.61)*	.74	41.11 (3.96)	.70
Ineffectiveness	57.25 (16.58)*	.75	44.69 (6.01)	.81
Perfectionism	57.73 (12.58)	.67	54.31 (10.51)	.55
Interpersonal distrust	53.73 (12.35)	.65	49.21 (10.00)	.60
Interoceptive awareness	59.34 (13.94)*	.69	45.72 (7.20)	.70
Maturity fears	60.00 (11.50)*	.55	52.82 (10.72)	.62
Asceticism	55.07 (15.53)*	.52	46.20 (8.71)	.56
Impulse regulation	61.22 (12.85)*	.70	46.56 (7.73)	.71
Social insecurity	57.59 (10.33)*	.55	48.03 (8.69)	.60

* Group with significant higher score in the variables

#### Differences in the level of anxiety (STAY)

Statistical differences on state and trait anxiety between groups [F(1,79) = 30.82; p = .0001] were found. No statistical differences between scales [F(1,79) = 1.26; p = .27] were found. The interaction between groups and STAI-Y scales seems to be insignificant [F(1,79) = .83; p = .37].

More specifically, post hoc test performed with Bonferroni adjustments confirmed that the group with moderate binging presented higher scores in state anxiety (p < .001) and trait anxiety (p < .001) than the group without binging. The group without binging (p > .05) and the group with moderate binging (p > .05) demonstrated the same level of state and trait anxiety.

#### Differences in the level of first order personality trait (EPQ)

Results revealed that participants (moderate binging vs. non-binging) did not differ significantly on personality trait [F(1,79) = 2.15; p = .15], but the interaction between groups and EPQ-R scales [F(3,237 = 13.25; p = .0001] and the interaction within EPQ-R scales [F(3,237 = 3.85; p = .01] were significant.

Post hoc test performed with Bonferroni adjustments showed that the group with moderate binging reported higher scores in psychoticism (p < .05) and neuroticism (p < .001) than the group of adolescents without binge eating. However, the group of adolescents without binge eating obtained higher scores in social desirability (p < .001) than the group with moderate binging. No statistical differences in extraversion (p > .05) between groups were found.

However, some differences were found comparing the personality traits separately in two groups. The group with moderate binging obtained lower scores in social desirability than psychoticism (p < .01), extraversion (p < .01), and neuroticism (p < .001).

#### Differences in the level of second order personality trait (IVE)

Regarding second order trait (IVE), results demonstrated statistical differences between groups [F(1,79) = 17.47; p = .0001] and in the interaction among groups and these aspects of personality [F(2,158) = 7.75; p = .001]. Even the interaction between impulsivity-venturesomeness-empathy was significant [F(2,158 = 7.16; p = .001].

Specifically, the group with moderate binging had a higher score in impulsivity than the group without binging (p < .001). No statistical differences between groups in venturesomeness (p > .05) and empathy (p > .05) were found.

In the group of adolescents without binge eating, no statistical differences within these trait were observed, while the group with moderate binging presented more impulsivity than venturesomeness (p < .001) and empathy (p < .01).

#### Differences in the levels of eating behaviors (EDI-2)

Results demonstrated statistical differences between groups [F(1,79) = 70.17; p < .001] such as interaction between groups and EDI-2 scales [F(10,790) = 4.70; p < .001]. The interaction between all EDI-2 scales was significant [F(10,790) = 6.04; p < .001].

In particular, post hoc tests performed with Bonferroni adjustments showed that the group with moderate binging tended to strive for thinness more than the group of adolescents without binge eating (p < .001). In addition to this, they showed a higher degree of bulimia (p < .001), body dissatisfaction (p < .001), ineffectiveness (p < .001), interoceptive awareness (p < .001), maturity fears (p < .01), asceticism (p < .001), impulse regulation (p < .001) and social insecurity (p < .001). No statistical differences between groups were found in perfectionism (p > .05) and interpersonal distrust (p > .05).

In Table 2 the simple comparisons were summarized within EDI-2 scales separately in moderate binging and in group of adolescents without binge eating. Only significant statistical comparisons within eating behaviors in both groups were reported. The group with moderate binging presented higher scores in bulimia and impulse regulation, while the group without binging reported higher scores in perfectionism and maturity fears (see Table 2).

**Table 2:** Differences between eating behaviors (EDI-2) in moderate and non-binging groups

	1	2	3	4	5	6	7	8	9	10
Moderate binging (*FD* = 28)										
Drive for thinness										
Bulimia	−4.79									
Body dissatisfaction	4.29	9.07*								
Ineffectiveness	1.88	6.67	−2.40							
Perfectionism	1.40	6.19	−2.88	−.48						
Interpersonal distrust	5.41	10.19*	1.12	3.52	4.00					
Interoceptive awareness	−.21	4.58	−4.49	−2.09	−1.61	−5.61				
Maturity fears	−.87	3.91	−5.16	−2.75	−2.28	−6.28	−.67			
Asceticism	4.06	8.85	−.22	2.18	2.66	−1.34	4.27	4.93		
Impulse regulation	−2.09	2.70	−6.37	−3.97	−3.49	−7.49	−1.88	−1.22	−6.15	
Social insecurity	1.54	6.33	−2.74	−.34	.14	−3.87	1.75	2.41	−2.52	3.63
Non-binging (*FD* = 53)										
Drive for thinness										
Bulimia	−3.37									
Body dissatisfaction	2.47	5.84								
Ineffectiveness	−1.11	2.27	−3.58							
Perfectionism	−10.72*	−7.35*	−13.19*	−9.62*						
Interpersonal distrust	−5.63	−2.26	−8.10*	−4.52	5.09					
Interoceptive awareness	−2.14	1.23	−4.61*	−1.03	8.58*	3.49				
Maturity fears	−9.23*	−5.86	−11.70*	−8.13*	1.49	−3.60	−7.09*			
Asceticism	−2.62	.76	−5.08	−1.51	8.11*	3.02	−.47	6.62		
Impulse regulation	−2.98	.40	−5.44*	−1.87	7.75*	2.66	−.83	6.26	−.36	
Social insecurity	−4.45	−1.07	−6.91*	−3.34	6.28	1.19	−2.30	4.79	−1.83	−1.47

Coefficient represent mean difference based on estimated marginal means

* p < .05

Unlike the group of adolescents with moderate binge eating, the group without binging reported lower scores in body dissatisfaction and the drive for thinness as compared with the other group.

### Correlation analysis and multiple regression analysis

Correlation analysis (Table 3) demonstrated that in both groups, state anxiety and trait anxiety are positively related with ineffectiveness, interpersonal distrust, interceptive awareness, impulse regulation and social insecurity.

**Table 3:** Correlations between personality characteristics and eating behaviors

	SA	TA	P	N	E	LIE	I	V	EM
Moderate binging (FD = 28)									
Drive for thinness	.23	.29	−.31	−.20	.02	.02	.06	−.33	.08
Bulimia	.32	.09	.13	.06	.28	−.18	.09	.04	−.14
Body dissatisfaction	.28	.22	−.31	−.08	−.03	.13	−.01	−.29	.40*
Ineffectiveness	.48**	.65**	−.36	−.35	.18	.15	−.31	−.41*	.34
Perfectionism	.31	.33	−.16	.03	.32	.09	.07	−.07	.06
Interpersonal distrust	.42*	.56**	−.09	−.37	.30	−.29	−.16	−.53**	−.08
Interoceptive awareness	.50**	.62**	−.17	−.21	.37	.36	.02	−.28	.27
Maturity fears	.12	.25	−.09	−.35	.03	.05	−.02	−.24	.07
Asceticism	.18	.35	−.38*	−.34	.08	.31	−.20	−.41*	.19
Impulse regulation	.39*	.46*	−.12	−.16	.52**	.11	−.20	−.07	.14
Social insecurity	.48*	.51**	−.08	−.33	.05	.01	−.18	−.26	.09
Non-binging (FD = 53)									
Drive for thinness	−.18	−.03	−.02	.02	.18	.03	.15	.24	.16
Bulimia	.14	.03	.24	−.24	.21	−.06	.06	.14	−.22
Body dissatisfaction	.17	.13	.10	−.24	.29*	−.15	.01	−.01	.04
Ineffectiveness	.39**	.45**	−.10	−.45**	.32*	.20	−.15	−.42**	.16
Perfectionism	−.13	−.23	−.14	.10	.07	.25	−.15	.05	.10
Interpersonal distrust	.36**	.35*	.16	−.50**	.44**	.07	−.10	−.33*	−.05
Interoceptive awareness	.50**	.63**	.17	−.30*	.35*	−.10	.15	.02	.15
Maturity fears	.18	.16	.00	.04	.19	−.01	.03	−.18	.25
Asceticism	.15	.13	.03	−.29*	.18	.22	−.13	.07	−.11
Impulse regulation	.41**	.49**	.36**	−.23	.52**	−.08	.39**	.00	−.10
Social insecurity	.27*	.348*	.10	−.323*	.507**	−.16	.14	−.06	.05

*SA* State-Anxiety, *TA* Trait-Anxiety, *P* psychoticism, *N* neuroticism, *E* extraversion, *I* impulsivity, *V* venturesomeness, *EM* empathy

*** p < .001;** p < .01;* p < .05

In the group with moderate binging (Table 3), the analysis showed no significant correlations between eating behaviors (recognized by the EDI-2 scales) and neuroticism. However psychoticism seems to be negatively related only to asceticism, while extraversion seems to be positively related only to impulse regulation. In this group, impulsivity did not relate to any eating behaviors, while venturesomess seemed to be negatively related with ineffectiveness, interpersonal distrust and asceticism. Empathy is positively related only to body dissatisfaction.

Conversely, in the group of adolescents without binge eating, psychoticism was positively related to impulse regulation, while neuroticism seems to be negatively related to ineffectiveness, interpersonal distrust, interceptive awareness, asceticism and social insecurity.

Moreover, in contrast to the group of adolescents with moderate binge eating, in the group of adolescents without binge eating, extraversion seems to be positively related to body dissatisfaction, ineffectiveness, interpersonal distrust, interoceptive awareness, impulse regulation and social insecurity. In this group, impulsivity is related to impulse regulation, while venturesomeness seems to be negatively related with ineffectiveness and interpersonal distrust. Empathy was not related to any eating behaviors (EDI-2 scales).

Multiple regression analysis determined the extent of the relationship between personality characteristics and eating behaviors (EDI-2 scales). In the group with moderate binging, trait anxiety seems to be relevant in ineffectiveness (t = 2.27; p = .01; β = .83) and interoceptive awareness (t = 2.13; p = .05; β = .65), while interpersonal distrust seems to be negatively influenced by venturesomeness (t = −2.23; p = .04; β = .49).

In the group of adolescents without binge eating, trait anxiety seems to be related to perfectionism (t = 2.63; p = .01; β = .60) and interoceptive awareness (t = 2.86; p = .007; β = .58). In this group, the neuroticism seems to be more relevant to perfectionism (t = 3.16; p = .003; β = .59), interpersonal distrust (t = −2.17; p = .04; β = .37) and social insecurity (t = 2.56; p = .01; β = .47), while the extraversion seems to be negatively related only to interpersonal distrust (t = −2.21; p = .03; β = .34). Moreover, impulsivity seems to be related to impulse regulation (t = 2.37; p = .02; β = .39).

## Discussion

The general aim of this study was to create an explorative snapshot of the characteristics that differentiate subjects with no symptoms of BED and subjects at risk for BED. The first hypothesis of this research was to analyze personality traits and eating behaviors (EDI-2 scales) in adolescents “at risk” of BED. For this reason, a group with moderate BED behaviors was compared to a group of adolescents that did not exhibit any binging behaviors.

### Differences between groups

The results of the present study are consistent with several studies showing that anxiety may have a relevant effect on eating behaviors (Goldfield and Legg 2006; Fitzsimmons-Craft et al. 2012) and confirmed the relevance of state and trait anxiety; in fact the group with moderate binging presented higher scores in the area of anxiety than the group of adolescents without binge eating.

As underlined in the introduction, many studies did not confirm the association between psychoticism and ED symptomatology and between extraversion and eating disorder (Wonderlich et al. 2009). Our results are only in part consistent with these conclusions: adolescents with moderate binging reported higher scores in psychoticism and Neuroticism than the group of adolescents without binge eating, while they did not differ in extraversion. However, it is necessary to emphasize that the group of adolescents without binge eating could be influenced by social desirability (they reported higher scores in the Lie scale).

These results demonstrate how psychoticism and neuroticism are two prospective risk factors for most common psychological problems (Eysenck and Eysenck 1993). Furthermore from a practical point of view this could help to directly target the maladaptive personality and match individuals to suitable intervention programs in psychology. For example, maladaptive characteristics may be targeted to help refine the treatment and prevention of binge eating disorder (Cassin and von Ranson 2005).

In line with Lyke and Spinella (2004), this research analyzed the differences in Impulsivity. Relevant differences were found between groups: the group with moderate binging reported a higher score in impulsivity as compared to the group of adolescents without binge eating. Venturesomeness and empathy were also observed. There does not seem to be any difference between the two groups. However, adolescents with moderate binging demonstrated more impulsivity than venturesomess and empathy than the group of adolescents without binge eating.

### Correlation between personality characteristics and eating behaviors

The second hypothesis of this research was to analyze the relationship between personality characteristics and eating behaviors (recognized by the EDI-2 scales) in adolescents “at risk” of BED. Positive associations between neuroticism and eating disorders have been demonstrated (Cassin and von Ranson 2005; Lyke and Spinella 2004; Cuzzocrea et al. 2012) and our results confirmed that in the group of adolescents without binge eating both neuroticism and psychoticism seem to be relevant aspects, such as extraversion, impulsivity and venturesomeness. However, the relation between personality characteristics and eating behaviors are different if we are looking at adolescents without binging behaviors or adolescents “at risk” of BED (moderate binging). In the group of adolescents with moderate binge eating, trait anxiety, extraversion, venturesomeness and empathy could play a more relevant role in the associations between psychoticism and ED symptomatology in university samples (Finlayson et al. 2002; Geissler and Kelly 1994; Janzen et al. 1993; Diaz-Marsa et al. 2000) than other personality characteristics. Specifically the lack of relationship between neuroticism and eating behavior in the group with moderate binge eating could be explained by the fact that in the group with moderate binge eating neuroticism could have a more direct effect in the development of specific binge eating behaviours than general eating behaviours; this is also demonstrated by the higher level of neuroticism in the group with moderate binge eating than the group of adolescents without binge eating.

The knowledge of these personality correlates of EDs could help to match individuals to suitable intervention programs. For example, individuals with EDs who are identified as being particularly prone to experiencing venturesomeness may benefit from treatment strategies that target this propensity directly. In short, understanding associations of personality and EDs has the potential to help refine the treatment and prevention of these disorders (Cassin and von Ranson 2005).

## Conclusion

Lilenfeld et al. (2006) described five conceptual models that could be most relevant to the relationship between personality and eating disorders. It is important to emphasize that the nature of these relationships has not been well defined yet.

It is our opinion that, if personality trait and eating behaviors (recognized by the EDI-2 scales) are related differently in different sampling, the predisposition model could be more credible. According to the predisposition model (Bruch 1973), a personality trait may cause a predisposition to an eating order, or at least increase the risk. These results seem to confirm the hypothesis that personality disturbance and eating disorders are independent conditions (Lyons et al. 1997).

Of course, this research alone cannot resolve such a complex question. But the fact that adolescents who did not present all criteria for a diagnosis of BED (but demonstrated some potential risk) showed differences in personality characteristics compared to adolescents without binge eating justifies the continuation of this type of research.

### Limitations and future implications

The results of this study represent a new stimulus to investigate thoroughly those aspects of personality that may be predictive of these disorders and to develop preventative strategies. It is our opinion that it is necessary to focus attention not only on clinical or non-clinical samples, but also on adolescents who could be considered at risk. It is necessary to point out that in this study the group with moderate binging could not be considered a clinical group, even if these subjects reported dysfunctional eating behavior. In fact a big strength of this study is that it focuses on the difference between “at risk” individuals and individuals without BED. Furthermore the use of a set of diversified personality traits provide the opportunity to have a broad view of the differences between the two groups.

The principal limit of this research was the difficulty in selecting participants: the sample was not large enough, but these first results justify future research in order to obtain more generalized data. Another important aspect to mention is that our results preclude any statements of causal relations and confirm that disordered eating as a complex and multifaceted phenomenon is difficult to simplify. Furthermore the sampling method and the sample characteristics (e.g.; diversity of the sample; the use of a sample from a university population) are factors that limit generalizability of the results. However, these results should make us reflect on the necessity of providing preventative intervention as soon as possible and to conduct well-designed trials (with a larger sample size and randomized allocation) of preventative intervention strategies.
